# Long-Lasting Olfactory Dysfunction in Hospital Workers Due to COVID-19: Prevalence, Clinical Characteristics, and Most Affected Odorants

**DOI:** 10.3390/ijerph19095777

**Published:** 2022-05-09

**Authors:** María Luisa Delgado-Losada, Jaime Bouhaben, Claudia Ruiz-Huerta, Marcelle V. Canto, Alice Helena Delgado-Lima

**Affiliations:** 1 Experimental Psychology, Cognitive Processes and Speech Therapy Department, Faculty of Psychology, Complutense University of Madrid, Campus de Somosaguas, 28223 Pozuelo de Alarcón, Spain; jaimebou@ucm.es (J.B.); alicedel@ucm.es (A.H.D.-L.); 2 Servicio de Medicina Preventiva, Hospital Universitario de la Cruz Roja, 28003 Madrid, Spain; claudia.ruiz-huerta@salud.madrid.org (C.R.-H.); mcanto@salud.madrid.org (M.V.C.)

**Keywords:** COVID-19, SARS-CoV-2 infection, olfactory dysfunction, anosmia, hospital workers, Sniffin’ Sticks

## Abstract

Hospital workers have increased exposure risk of healthcare-associated infections due to the frontline nature of their work. Olfactory dysfunction is highly prevalent. The objectives for this investigation are to study the prevalence of long-lasting olfactory dysfunction associated with COVID-19 infection in hospital workers during the first pandemic wave, to identify clinical characteristics and associated symptomatology, and to analyze how many patients with COVID-19 infection had developed olfactory dysfunction during infection and maintained a reduced olfactory function for approximately 10 weeks after diagnosis. Between June and July of 2020, a cross-sectional study was carried out at the Hospital Central de la Cruz Roja San José and Santa Adela in Madrid, Spain. One hundred sixty-four participants were included, of which 110 were patient-facing healthcare staff and 54 were non-patient-facing healthcare staff. Participants were split into three groups, according to COVID-19 diagnosis and presence of COVID-19 related olfactory symptomatology. Participants were asked to complete a structured online questionnaire along with Sniffin’ Stick Olfactory Test measurements. In this study, 88 participants were confirmed for COVID-19 infection, 59 of those participants also reported olfactory symptomatology. The prevalence of COVID-19 infection was 11.35%, and the prevalence for olfactory dysfunction was 67.05%. Olfactory dysfunction associated with COVID-19 infection leads to long-lasting olfactory loss. Objective assessment with Sniffin’ Stick Olfactory Test points to odor identification as the most affected process. Lemon, liquorice, solvent, and rose are the odors that are worst recognized. Mint, banana, solvent, garlic, coffee, and pineapple, although they are identified, are perceived with less intensity. The findings of this study confirmed a high prevalence of SARS-CoV-2 infection among the hospital workers.

## 1. Introduction

Coronavirus disease 2019 (COVID-19) is caused by the Severe Acute Respiratory Syndrome Coronavirus 2 (SARS-CoV-2). The most common symptoms are fever, fatigue, cough, dyspnea, myalgia, arthralgia, diarrhea, and headache. Bilateral pneumonia is frequently seen on chest radiographs or computed tomography scans, along with bilateral pulmonary involvement and bilateral pneumonia [1,2].

Angiotensin-converting enzyme (ACE2) is the functional receptor for SARS-CoV-2 [3]. Given that the respiratory epithelium is the main site of SARS-CoV-2 and many other viruses, it is not surprising that COVID-19 affects the olfactory neuroepithelium [4,5]. Sudden olfactory loss, anosmia (complete loss of smell), or hyposmia (partial loss of smell) have been reported as symptoms of COVID-19, presenting as an initial symptom, concomitantly or immediately after general symptoms [6,7,8]. In some cases, anosmia or hyposmia may be the first and only symptom, and may or may not be associated with taste disturbances [9,10,11].

In addition, many patients report parosmia (distorted smell in the presence of a familiar odor source) and phantosmia (experience of smell in absence of an odor source) development [12]. These alterations are recognized to have a particularly pronounced impact on quality of life as most experiences involve unpleasant smells (malodours) [13]. The “COVID smell” is generally an unpleasant, chemical-type burning smell. The most common triggers are coffee, onions, garlic, meat, and citrus fruits, along with toiletries such as toothpaste, but the underlying mechanism for these phenomena is unclear [14,15].

Incidence of smell loss in COVID-19 infection reported by studies indicates a great variability because the methods used to describe clinical findings have been different. Most studies are generally based on self-reported data or unvalidated questionnaires, and studies based on objective evidence are rare. Speth et al. [10] studied the loss of smell via questionnaire with a group of 103 participants positive for COVID-19, where 95% of the participants reported olfactory alterations. Menni et al. [16] conducted a study with a community survey through a mobile phone application. In the international study by Parma et al. [17], a multilingual questionnaire was applied to assess the quantity and quality of self-reported perception in three different chemosensory modalities (smell, taste, and chemosynthesis). Eleven countries, including Spain, collaborated in the application. A study carried out at the Hospital Clínico San Carlos with hospitalized patients, Gómez-Iglesias et al. [18] indicate that 97.7% of patients with COVID-19 presented olfactory alterations and 92.95% reported taste alterations that could be explained by loss of retronasal olfactory perception.

Lechien et al. [6] applied the Sniffin’ Sticks Olfactory Test and observed that 52% of the patients had anosmia and 24% hyposmia. Moein et al. [19] applied the University of Pennsylvania Odor Identification Testa and found that 98% of patients positive for COVID-19 had anosmia or hyposmia.

The combination of self-reported information through online questionnaires such as those used in the aforementioned studies, together with an objective evaluation test for olfactory performance, allows for more robust studies and therefore leads to better results [20].

Although the exact pathophysiology of olfactory dysfunction (OD) in COVID-19 is not well understood, anosmia appears to be short-lived and self-resolving. Research indicates that recovery of olfactory perception occurs parallel with recovery from the infection [21,22,23]. This improvement over time would suggest a competitive action of the virus on olfactory cell receptors or local inflammatory phenomena, rather than permanent damage to the olfactory neuroepithelium [24].

Studies related to loss of smell in COVID-19 patients describe their recovery in the first weeks [25], with most patients regaining their sense of smell within 14 days of resolution of COVID-19 infection (72.6% within 8 days), and only 3.3% of the cases with hyposmia, and 3.4% with anosmia, took more than 15 days [6,22,26,27,28,29]. Although a large proportion of people recover smell perception within weeks, more than 10% report persistent problems including anosmia (loss of smell), hyposmia (reduced smell), parosmia, and phantosmia [17]. However, in general, all these studies are self-reported data [23,30]. In a study by Boscolo Rizzo et al. [31], a significant mismatch was found between self-reported olfactory function and objective assessment with olfactory evaluation tests.

Hence, it is of interest to study olfactory dysfunction through objective assessment tests using validated tests in the population to which they are to be applied, complementing the information with subjective perception declared by the patients, and to analyze the gradual recovery and long-lasting evolution of OD in patients with COVID-19 infection who extend their investigation beyond the first few weeks.

Hospital workers (HWs) (patient-facing and non-patient-facing hospital staff) are at the frontline in the care of patients, but few studies have focused on studying the progression of the disease among them. And there is little data available regarding the prevalence of infection in HWs and close to none featuring their clinical, symptomatology, and OD associated with the infection. HWs are a high-risk group for contracting SARS-CoV-2 infection from patients, visitants, or other colleagues, due to their close contact [32,33,34]. In China, more than 2000 cases among HWs were infected by February 2020 [35]. In March 2020, Italy reported 2600 HW infections [36]. In Spain, from the beginning of the pandemic to May 11 of 2020, 40,921 cases were reported to the national epidemiological surveillance network, and in February 2022, more than 150,000 confirmed cases among staff have been reported [37].

Due to work-related risks, patient-facing healthcare staff are daily exposed to increased viral loads, which may lead to different expressions of chemosensory disorders, both in terms of prevalence, severity, and/or rate of recovery. In the United States, in a study by Kaye et al. [38], one third of the hospital workers who tested positive for COVID-19 infection had anosmia, and in a study by Kempker et al. [39] the number reached 51%. In Europe, a Belgian study disclosed that 40% of healthcare personnel reported losing their sense of smell [40], while a Danish study with a larger sample found OD in 32.4% of patient-facing healthcare staff [41].

As far as we know, in Spain there are few studies on the prevalence of anosmia in HWs, except for some inconclusive studies for their small case series [42,43,44].

The objectives for this investigation are (i) to study the prevalence of long-lasting olfactory dysfunction associated with COVID-19 infection in hospital workers during the first pandemic wave, (ii) to identify clinical characteristics and associated symptomatology, and (iii) to analyze how many hospital workers with COVID-19 infection had developed olfactory dysfunction during infection and maintained a reduced olfactory function for approximately 10 weeks after diagnosis. 

## 2. Method

### 2.1. Participants

A total of 184 participants were initially recruited. This initial sample was composed of workers from Hospital Central de la Cruz Roja San José y Santa Adela (HCCR) (Madrid, Spain). Recruitment was performed through advertisements within hospital facilities. From this initial pool, 20 participants were excluded due to eligibility criteria. Thus, the final sample was composed of 164 participants (110 patient-facing healthcare staff and 54 non-patient-facing hospital staff). A participants’ flow diagram is available in Supplementary Material S2. The HCCR is a public hospital, belonging to the Health Service of Madrid, SERMAS, and it has 156 beds and 775 workers. It is considered a support hospital, i.e., it offers its health services to the citizens of the Community of Madrid in two main verticals: elderly attention and care and surgery of low-medium complexity processes. Thus, the participation rate in this study was almost 25%.

Inclusion criteria of the study were: (i) to be 18 years or older, (ii) absence of current otorhinolaryngology alterations, previous to COVID-19 diagnosis (reported by participant), (iii) being an active hospital worker during the initial stage of the COVID-19 pandemic (February–April, 2020), (iv) 10 weeks have passed since a positive COVID-19 RT-PCR test to the time of the assessment for the SARS-CoV-2 infected HWs group with olfactory symptoms, and (v) compliance with testing procedure. Exclusion criteria were: (i) a medical history of olfactory alterations, including nasal polyposis, sinusitis, or prior nasal surgery, (ii) medication intake with repercussion in olfactory performance (such as some antibiotics, antiepileptics, antithyroids, benzodiazepines, or antiarrhythmics), (iii) presence or suspicion of cognitive impairment and/or neurologic or psychiatric dysfunctions, and (iv) self-declared pregnancy.

All COVID-19 diagnoses were performed via nasopharynx RT-PCR testing between March and April of 2020, or via IgG (CLIA) antibodies detection, performed in June and July of 2020 in all HWs, which allowed detection in asymptomatic cases. All testing was performed with the highest regard for patients’ and examiners’ safety, using appropriate personal protective equipment.

Participants were split into three groups, according to COVID-19 diagnosis and presence of COVID-19 related olfactory symptomatology. Therefore, the three groups were: (i) participants who were diagnosed with COVID-19 infection and reported olfactory symptoms, (ii) participants who were diagnosed with COVID-19 infection but without olfactory symptoms, and (iii) control participants, also HW, who were not infected with COVID-19.

All participants were assessed with the complete version of the SSOT. In addition, they filled out an online questionnaire so that clinical and demographic variables could be collected. Data collection took place between the end of June and the end of July, 2020.

### 2.2. Measures and Testing Procedure

Sniffin’ Sticks Olfactory Test (Spanish version): The original Sniffin’ Sticks Olfactory Test (SSOT) (Burghart Messtechnik GmbH, Wedel, Germany) was adapted to the Spanish population by Delgado-Losada et al. [45]. The complete version includes three tests that aim to measure different components of olfactory function, namely, olfactory threshold (T), odor discrimination (D), and odor identification (I). Each test gives a unique score, ranging from 0 to 16, representing each olfactory component, and it may also be administered independently. The sum of the three odor scores (T, D, and I) defines a composite score (TDI, ranging from 0 to 48), which measures general olfactory function. Olfactory function was assessed for both nostrils. For odor presentation, pens with a length of 14 cm and a diameter of 1.3 cm were used. Each pen was filled with 4 mL of the corresponding liquid odorant. The evaluator took the pen’s cap off and put the tip of the pen in front of the participant’s nostrils, with an approximate distance of 2 cm. In any case, the tip of the pen never physically touched the participant’s nose. In all of the three tests, each odor pen was presented to the participant for 3 s. The overall time of administration ranged from 30 to 45 min, depending on how long the T subtest lasted.

The order of test presentation is T, D, I, as in the original version [46]. The administration procedure follows the one established in this original version. However, it includes the procedure tested in Delgado-Losada et al. [47], which results in three I scores: free recall score, recognition score, and subjective intensity score, included as a visual analog scale (VAS), to assess the perceived intensity of each odorant (Supplementary Material S1). The use of the VAS scale allows assessing the intensity with which the participants perceive odors through an ordinal quantitative scale of their sensory dysfunction. Recognition score is the one that composes the TDI, following the original version.

In addition, the original test set the 10th percentile as the cutoff point for hyposmia [46]. These criteria are also taken into account in this present study. Spanish validation reports the 10th percentile in different age cohorts for each score, as well as for TDI [45]. Smell loss measure, a dichotomous measure (yes/no), was obtained by classifying participants above or below TDI’s 10th percentile.

Participant testing was performed in a quiet, well-ventilated room to avoid any background smell interfering with the test odors and with the use of odorless gloves. All participants were told not to eat, drink, smoke, chew gum, put on cologne, or brush their teeth up to 1 h before participating in the test (they could drink water).

Clinical Questionnaire: Participants were instructed to fill out an online questionnaire survey based on Parma et al. in order to collect demographic and clinical information related to health, habits, and COVID-19 symptomatology prior to olfactory assessment [17]. Participants who tested positive for COVID-19 infection were also asked to score their self-perception of olfactory performance before and after the disease. Current nicotine or alcohol usage was also taken into consideration.

### 2.3. Study Design

The present study follows a cross-sectional quasi-experimental design, as the main independent variable was set by researchers, but no random allocation was possible. This independent variable was a combination of presence of COVID-19 infection and COVID-19 with or without olfactory symptomatology. As a result of this combination, three categories were established: COVID-19 diagnosis with olfactory symptoms, COVID-19 diagnosis without olfactory symptoms, and healthy controls (no COVID-19 infection). As pointed out in the Measures and Testing Procedure section, COVID-19 testing was performed via RT-PCR or via IgG (CLIA) antibodies detection. COVID-19-related olfactory symptomatology was reported by participants.

Olfactory assessment was performed between June and July, 2020 on each participant. COVID-19 participants with olfactory symptomatology were assessed approximately 10 weeks after COVID-19 diagnosis via RT-PCR. COVID-19 symptoms were reported by infected participants, and this information was collected through the online clinical questionnaire. As it was performed several weeks after COVID-19 infection, objective olfactory assessment allows one to obtain a long-lasting smell loss measure.

According to the Measures and Testing Procedure section, six dependent variables were obtained from the administration of SSOT (Spanish version): T score, D score, Identification–Free recall score, Identification–Recognition score, Identification–Subjective intensity score, and TDI score. Further, TDI score was taken into account to classify participants’ olfactory performance in normosmic (above 10th percentile) or hyposmic (equal or below 10th percentile) [46]. This dichotomous classification criteria intends to measure long-lasting smell loss.

This study was ruled by the principles of the Declaration of Helsinki (Edinburgh, 2013) and was approved by the Ethics Committee from University Hospital San Carlos (Madrid, Spain) (ref. number: 20/515-E). Every participant was informed about the study objectives and signed an informed consent prior to the assessment. The study was adjusted to standards of good clinical practice (art.34 RD 223/2004; community directive 2001/20/CE), and to the protection of personal data and confidentiality (European Data Protection Regulation, and in accordance with the Organic Law 3/2018 on the Protection of Personal Data and Guarantee of Digital Rights).

### 2.4. Statistical Analyses

The entire statistical analyses were performed with R software, version 3.5.2 [47]. At first, the prevalence (P) of COVID-19 infection was computed as the quotient of cases for the total of HWs. This prevalence was also computed for healthcare and non-healthcare hospital workers. The prevalence for olfactory symptomatology was also computed within the COVID-19 positive subsample. In addition, participants were classified as hyposmic or normosmic based on their SSOT TDI score (above or below 10th percentile). In order to check whether COVID-19 infection and olfactory symptomatology might be risk factors for long-lasting smell loss, odds ratios with their respective 95% confidence intervals were estimated.

Later, descriptive analysis was performed. Mean and standard deviation were computed for each COVID-19 group in each clinical and demographic variable. Plus, F-tests or chi-squared tests were estimated in order to test potential statistical differences between COVID-19 categories. Means and standard deviations of SSOT measures, i.e., objective olfactory performance measures, are also reported.

After that, ANCOVA models were adjusted for each olfactory measure as a dependent variable, with COVID-19 categories as independent variables. Age and Smoking were introduced in these models, due to their potential confound effects. Gender was not introduced as covariate because no relation was found in our previous validation studies of the SSOT, Spanish version [45,48]. Although females predominate the overall sample, they are equally distributed between groups (chi = 0.054, df = 1, *p* = 0.973). As multiple ANCOVA models are estimated, the alpha level is set to 0.008 (lower than 0.05), in order to prevent increment of type I error’s probability (Bonferroni correction). Post-hoc multiple comparisons tests between COVID-19 categories were applied to each statistically significant ANCOVA model. Due to imbalanced data, unequal variances were assumed. In this analysis, only corrected *p*-values under false discovery rate [49] were considered.

Finally, the proportions of right answers per item of Identification–Recognition within the two COVID-19 subsamples (with and without olfactory symptoms) were obtained, and chi-squared tests were applied. Average scores per item of Identification–Subjective intensity in these two subsamples, and *t*-tests (under Welch’s correction) were performed.

## 3. Results

Among the 164 participants included, 110 (67.07%) were patient-facing healthcare staff, i.e., 42 (25.61%) physicians, 40 (24.39%) nurses, 20 (12.20%) nursing assistant, and 8 (4.88%) other health care workers (physiotherapists, occupational therapists, or psychologists), and the remaining 54 (32.93%) were hospital staff, i.e., 10 (6.09%) orderlies, 39 (23.78%) administrative staff, and 5 (3.05%) other non-patient-facing healthcare staff (cleaning or laundry staff).

Regarding the participants, 88 were confirmed for COVID-19 infection; 59 of those participants also reported olfactory symptomatology. Consequently, COVID-19 prevalence regarding HWs (*n* = 775) is *p* = 0.1135 (11.35%). The prevalence of COVID-19 infection varies between healthcare and non-healthcare hospital workers. From these 88 workers positive for COVID-19 infection, 65 were healthcare professionals (physicians, nurses, nursing assistant, and others), so prevalence was *p* = 0.0837 (8.37%), while the prevalence among non-healthcare hospital workers was *p* = 0.0296 (2.96%). Regarding the COVID-19 groups (with and without olfactory symptoms), olfactory symptomatology prevalence was *p* = 0.6704 (67.04%), within a time interval of approximately 10 weeks. Long-lasting objective smell loss was evaluated with the SSOT TDI score. Within the control group (*n* = 76), 12 were classified as hyposmic (*p* = 0.1875), while in the COVID-19 groups, 27 participants were considered as hyposmic (*p* = 0.3069). Thus, odds ratio (OR = 2.334, 95% CI = [1.102, 5.201]) indicates that COVID-19 infection increases the odds for long-lasting smell loss. Focusing on olfactory symptomatology during COVID-19 infection, 5 of 29 participants without olfactory symptoms were classified as hyposmic (*p* = 0.1724), while 22 of 59 participants who reported olfactory symptoms during COVID-19 infection were classified as hyposmic 10 weeks after COVID-19 infection (*p* = 0.3728). COVID-19 infection by itself does not increase the odds of long-lasting smell loss (OR = 1.126, 95% CI = [0.321, 3.445]), but COVID-19 infection with olfactory symptoms significantly increases the odds of long-lasting smell loss (OR = 3.127, 95% CI = [1.4, 7.276]).

**Figure 1:**
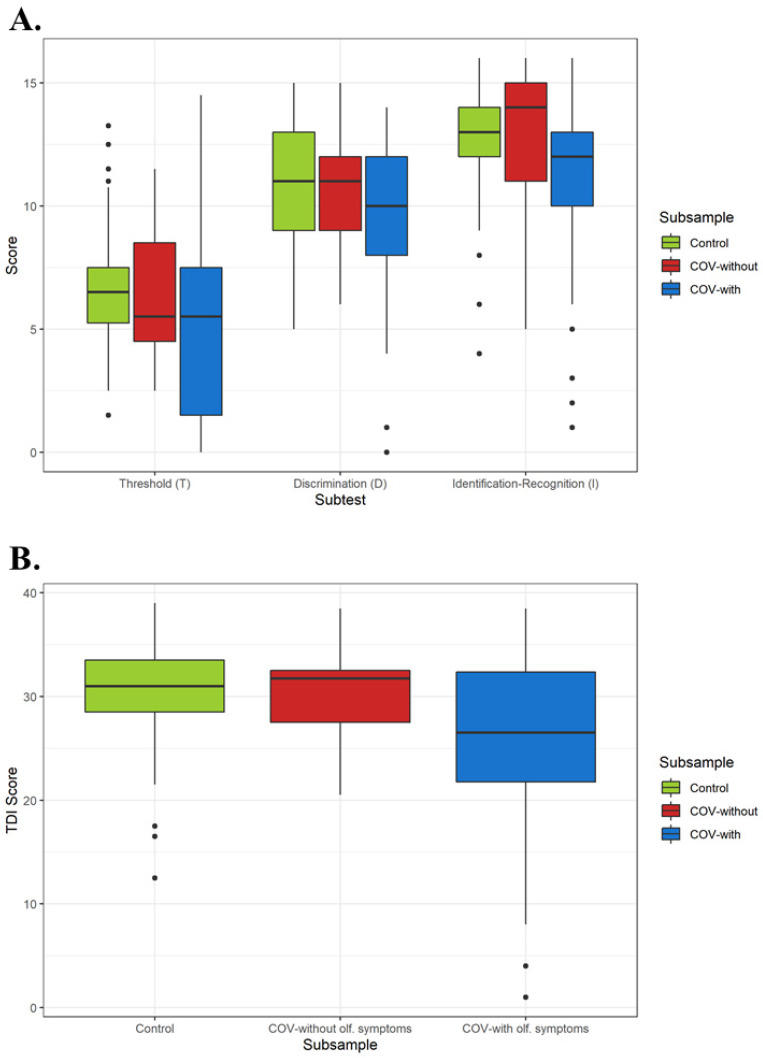
Boxplots by COVID-19 subsample and control group for: (**A**) T, D, and I scores (I-Recognition) and (**B**) TDI score.

Descriptive analysis is shown in Table 1. Potential differences between olfactory groups in demographic and clinical covariates were tested. Statistically significant differences were found in relation to Age. No other differences between COVID-19 categories were found regarding clinical and demographic variables. Concerning COVID-19 subgroups, Table 2 describes their SARS-CoV-2-related symptomatology. Common olfactory symptoms that were found in COVID-19 olfactory subsample were smell loss or decrease (prevalence of 1) or changes in food taste (prevalence of 0.729). Other unusual symptoms such as stuffy nose (prevalence of 0.356), parosmia, and phantosmia were reported by participants. The prevalence of parosmia was 0.44, while the prevalence of phantosmia was 0.1186. Figure 1 shows (a) T, D, and I scores (I-Recognition) and (b) TDI score, by COVID-19 subsamples and control group.

ANCOVA analyses were applied to each olfactory domain as a dependent variable, with COVID-19 infection as an independent variable and adjusting by Age and Smoking. Interaction effects between covariates and the independent variable were also tested, but no statistically significant interaction effect was identified from the analysis. These results are summarized in Table 3. The Bonferroni method was applied to correct alpha levels because of the multiple comparisons. The corrected alpha level was set to 0.008.

The main consequences of COVID-19 olfactory symptomatology were reflected in Identification–Recognition score (F = 6.811, df = 2, *p* = 0.001) and TDI score (F = 8.233, df = 2, *p* = 0.0004), after alpha correction. This effect was also found, but uncorrected, in the Threshold and Identification–Subjective Intensity scores. Post-hoc comparisons between COVID-19 infection categories were also performed. No significant differences between COVID-19 groups were found, either in T scores or D score (*p* > 0.05), so these effects in their respective ANCOVA model were not taken into account. In the Identification–Free recall score, significant differences were found between No-COVID-19 and COVID-19 groups with olfactory symptoms groups (*p* = 0.027), but there were no evidence to point differences between No-COVID-19 and COVID-19 without olfactory symptoms or between COVID-19 groups (*p* > 0.05). Next, in Identification–Recognition, No-COVID-19 and COVID-19 with olfactory symptoms groups had statistically significant different means (*p* = 0.002). In addition, COVID-19 without olfactory symptoms group scored significantly higher than COVID with olfactory symptoms group (*p* = 0.002), showing better olfactory performance. No differences were found between No-COVID-19 and COVID-19 without olfactory symptoms group. Regarding Identification–Subjective intensity score, only statistically significant differences between the no-COVID-19 and the COVID-19 with symptoms group were reported (*p* = 0.031), implying higher odor intensities for No-COVID-19 participants. Finally, in TDI score, post-hoc comparisons were similar to Identification–Recognition. Average olfactory performance was no different between no-COVID-19 and COVID-19 without symptoms groups (*p* > 0.05). Olfaction in COVID-19 participants who reported olfactory symptomatology was, on average, lower than participants with no-COVID-19 (*p* = 0.001) and with COVID-19 but with no olfactory symptomatology (*p* = 0.001).

**Figure 2:**
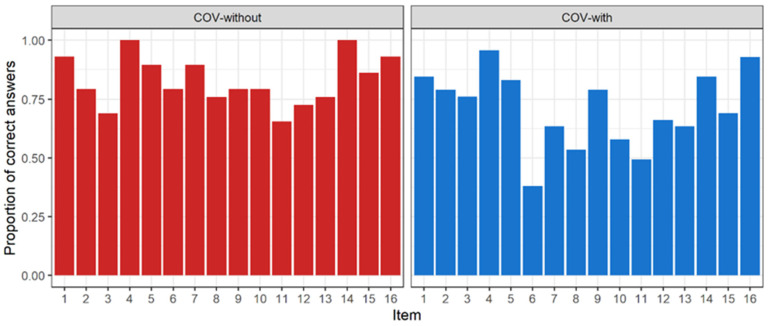
Proportion of right answers per odor in Identification (I) subtest (Recognition score).

Furthermore, Table 4 and Figure 2 show the percentages of success per item in Identification–Recognition score by COVID-19 symptomatology, alongside their respective proportion tests (chi-squared). Table 5 shows average subjective intensity per odorant in Identification–Subjective intensity.

Proportion of right answers is statistically higher in the non-olfactory COVID-19 group for items 6, 7, 8, and 14. These odorants are identified as lemon, licorice, solvent, and rose, respectively. On the other hand, subsample with no olfactory symptomatology also scored higher in subjective intensity for items 4, 5, 8, 9, 10, and 13. These odorants are identified as peppermint, banana, solvent, garlic, coffee, and pineapple, respectively, which are the odorants the participants had more difficulty in identifying.

## 4. Discussion

To our knowledge, this is the first report in Spain on the prevalence of long-lasting olfactory dysfunction associated with COVID-19 in HWs. This study examined the clinical characteristics and associated symptomatology of workers infected with SARS-CoV-2. Olfactory performance was analyzed approximately 10 weeks after diagnosis using a combined method that consisted of a self-completed online questionnaire and the application of the SSOT, an instrument for evaluating olfactory capacity, validated in Spain by our group. Olfactory performance data from SARS-CoV-2 infected HWs were compared with a control group of workers from the same hospital.

Our results reveal a global prevalence of COVID-19 of 11.35% among HWs, a similar figure to those published in our country by large acute care hospitals. For example, Hospital Clinic de Barcelona reports a prevalence of 11.2% [33] and Hospital 12 de Octubre in Madrid 11.6% [50]. However, it is higher than those found in other studies, such as the investigation by Moreno Borraz et al. [51] carried out at the San Juan de Dios Hospital in Zaragoza, a medium and long stay hospital, similar in care capacity to the HCCR, where, in a group of 294 HWs, they found a prevalence of 8.16%. Additionally, similar results were found in a study by Garcia-Basteiro et al., who, in a sample of 578 participants from a tertiary hospital in Barcelona, found a prevalence in HWs of 9.3% [33], higher than the prevalence found in Barcelona’s general population (6.8%) [52]. Self-reported loss of smell underestimates the true prevalence of olfactory dysfunction compared to when objective tests are used, as shown by Moein et al. [19]. In general, the oscillation of the prevalence of SARS-CoV-2 infection varies between 0.4% in the study by Olalla et al., in 498 Spanish hospital workers [53], and 57.06% in the study by Breazzano et al. [54], conducted in New York. The great variability is fundamentally due to the diversity of methodologies used in the studies.

In terms of prevalence in professional categories, there are conflicting results in the literature identifying the job category associated with highest risk of COVID-19 infection among HWs [55,56,57]. Studies generally analyze prevalence by comparing different departments [58,59,60] or by profession, between doctors and nurses [56,57], but there are few comparative studies between healthcare and non-healthcare professionals. In our study, the prevalence by groups was 8.37% for patient-facing healthcare staff compared to 2.96% who were not patient-facing healthcare staff. Our data is similar to those found by Moreno Borraz et al. [51], who found a prevalence by groups of 8.91% in healthcare professionals compared to 4.26% in non-healthcare professionals. It might be that healthcare professionals are better at reporting symptoms due to their own training.

Regarding the smell loss in HWs due to COVID-19 infection, our results indicate a prevalence of 67.05%. This result is higher than those found in other investigations carried out in Spanish hospitals, such as Deschamps-Perdomo et al. [44], who point out that 37.25% of workers presented loss of smell perception, although it is lower than those found in the Spanish multicenter study by Varona et al. [61], where 77.4% of HWs diagnosed with COVID-19 infection had loss of smell perception. In the study by Andrews et al. [62] with 114 HWs, a prevalence of 73.1% was found. In general, the prevalence of studies outside our country indicates a figure between 14.4% and 79% [39,40,41]. The high number of HWs (doctors, nurses, and nursing assistants) who participated and tested positive for SARS-CoV-2 in our study could explain the high prevalence found. Various studies with HWs, such as the ones by Clemency et al. [63] or Rudberg et al. [64], indicate that loss of smell perception was the symptom with the highest positive likelihood ratio for SARS-CoV-2 infection, and one of the symptoms chosen as the most sensitive indicator to refer people to diagnostic tests.

If we compare the prevalence of olfactory dysfunction of the HWs in our study with the prevalence found in the general population, we observe that ours is higher. Thus, for example, in a Spanish multicenter study by Beltrán-Corbellini et al. [26], 35.5% of the participants had loss of smell perception. Additionally, the first seroprevalence study carried out in Spain indicated that the prevalence for anosmia in patients diagnosed with COVID-19 was 43.3% [65]. Our results are consistent with those found in other studies that showed that HWs are a group at high risk of exposure to the virus as they are the first line of response to the COVID-19 pandemic [35,36,66,67]. These findings were also observed in other zoonotic coronavirus outbreaks (SARS and MERS), when a substantial proportion of the infected population were HWs [68].

Among the symptomatic COVID-19-positive HWs, in this study, the most frequently reported symptoms were: loss of smell perception (67.05%), headache (48.86%), fatigue (44.32%), myalgia (43.18%), dry cough (36.36%), and diarrhea (44.07%). Associated with olfactory dysfunction, of the 59 HWs, 48.86% report experiencing changes in food taste and 44.07% loss of appetite. In this symptom, we found statistically significant differences between the group of patients who had loss of smell perception and those who did not. Our results are in agreement with those found in studies in which anosmia or hyposmia, headache, fatigue, myalgia, and cough were reported as symptoms associated with the diagnosis of COVID-19 infection [9,16,68,69]. Thus, the symptoms described in our series are similar to those found in other published studies, except for anosmia, which was present in 67.05% of participants, a higher percentage than in the majority of studies.

Identifying and understanding the risk factors and clinical characteristics associated with SARSCoV-2 infection among HWs are of paramount importance to mitigate the spread of the virus in hospital settings, for high-risk patients and other HWs.

Regarding sgender and age of the HWs with SARS-CoV-2 infection, in our study 83.05% of the HWs with olfactory dysfunction were women, with a mean age of 44.7 years. Similar results were found in other studies such as the one by Andrews et al. [62] in which 75.4% of HWs who experienced loss of smell perception were women with a mean age of 38 years, and the same occurs in the overall population [9,24,27]. Although various theories have been suggested to try to explain why women present OD more frequently than men [70], the reason for these findings still remains unclear.

The groups of HWs in our study are mostly healthy professionals; both HWs with and without COVID-19 infection have few comorbidities (allergies, hypertension, obesity, cardiac, neurological or respiratory issues). Our results are consistent with those of Paderno et al. [71], who carried out a cross-sectional analysis in which the prevalence of OD was compared in hospitalized versus non-hospitalized COVID-19 patients, it was observed that OD predominated in younger patients, in women, and in those without comorbidities. In general, loss of smell perception is more common in women, younger age groups, and in healthier individuals. It may be associated with lower mortality and a milder disease trajectory compared to the general cohort.

Interestingly, significantly fewer smokers were found in our group of participants diagnosed with COVID-19 than in the control cohort. Our results are in line with those found in other studies in Iran [19], China [72], and the USA [73]. Recent studies indicate that smoking upregulated ACE-2 expression in the airways, potentially predisposing to increased risk of coronavirus infection, but paradoxically protecting the host against acute lung injury [74]. Future research is needed to clarify this possible protective mechanism of tobacco against SARS-CoV-2 infection.

Our study also indicates 26 cases of parosmia and 7 of phantosmia in the HWs, i.e., 44.07% and 11.86% of participants who reported loss of smell perception associated with diagnosis of COVID-19 infection. These results are superior to those found by Lechien et al. [9], with a sample of 417 confirmed COVID-19 patients, which found cases of parosmia and phantosmia in 32.4% and 12.6% of patients, respectively. They are also higher than those found by Vandersteen et al. [75] in a sample of 54 confirmed COVID-19 patients who found 20.4% cases of parosmia and 24.1% of phantosmia. We think that this may be due to the characteristics of the participants, being hospital professionals, they have a greater knowledge of the symptoms associated with COVID-19 infection and a greater ability to recognize these symptoms, no matter how mild they may be, and to report them. However, we have not found any studies in HWs indicating cases of parosmia and phantosmia. The underlying mechanism of parosmia and phantosmia is unclear [12]. One theory is that the decreased number of functional olfactory neurons in the olfactory epithelium leads to incomplete characterization of odorants [76].

Another strength of this research is the administration of the SSOT to assess olfactory performance. The TDI score is an estimator for general olfactory ability. The results show that the group of patients who presented symptoms of OD during their SARS-CoV-2 infection scored significantly lower than the group of patients without olfactory symptoms and the controls. Based on the results of the different subtests of the SSOT, we found that the group of participants with OD scored on average lower than the other two groups. However, these differences between groups are only statistically significant in the Identification–Recognition scores (corrected for multiple comparisons). Although the difference in the ANCOVA model for Identification–Subjective intensity does not reach significance under *p*-value correction, corrected post-hoc comparisons between groups do show relevant differences between groups.

Therefore, these results seem to indicate that odor identification is the most affected process after COVID-19 infection with olfactory symptoms. This is in line with other studies that have demonstrated that the Identification–Recognition subdomain is predominant in post-COVID [77,78].

In line with the above, a significant result in our study that has not been published before, to our knowledge, is related to the odors that patients with olfactory loss due to COVID-19 infection have the greatest difficulty in identifying. These results indicate that lemon, liquorice, solvent, and rose are the odors that are worst recognized. In the same way, in this group we have detected that there are odors that, although they are identified, they are perceived with less intensity. Namely, mint, banana, solvent, garlic, coffee, and pineapple. We would like to highlight that the cultural factors associated with these odors in our population have been previously controlled by the validation of the SSOT carried out by our research group, and all the odors that have been mentioned above present a level of familiarity greater than 75% [47]. Studies indicate that these odors are considered primary odors based on the size and shape of their molecules [79,80]. Having other studies on this topic would be interesting to support or confront our results.

Regarding the duration of olfactory loss in COVID-19 infection, it is still not clear. In our study, we have been able to demonstrate with an olfactory test validated in our country [45,48] that the olfactory dysfunctions associated with COVID-19 infection last and are detectable weeks after the onset of symptoms. Studies suggest that OD will improve in a substantial fraction within 1 to 2 weeks in conjunction with improvement of infection [6,22,26,27,28,29]. Although a large proportion of people recover smell perception within weeks, more than 10% report persistent problems [17]. Andrews et al. found that complete recovery occurred within a median of 52 days in 31.8% and was maintained in 68.2% of subjects [62]. More studies on long-lasting OD are needed.

Our study has several strengths, one of those being the usage of a combined methodology where all participants completed an online questionnaire declaring symptoms of SARS-CoV-2 infection, previous illnesses, and detailed description of their olfactory symptoms together with objective measurements of olfactory performance. Furthermore, having three groups of participants, professionals without COVID-19 and those with COVID-19, and within this last group, participants with and without OD, brings rigor to our work. Another strength is its capacity to study the prevalence of long-lasting smell loss, and not only in healthcare professionals who cared for patients on the front line during the first wave of the pandemic. It also provides a detailed analysis of the odors that are worst recognized and identified by the group of HWs with loss of smell.

However, it also has some limitations. The study was carried out in a single hospital, which does not allow the results to be generalized to other health contexts. In addition, we have to consider the possible existence of a recall bias on the part of the HWs when they completed the online self-recognition symptoms questionnaire and the olfactory self-assessment retrospectively. We do not have olfactory measurements prior to the COVID-19 outbreak, so we cannot rule out the possibility that some had olfactory dysfunction beforehand. Additionally, we have a single evaluation and not a longitudinal study, so our claims are limited to the time of testing.

In general, more research is needed to: (i) find out if the clinical characteristics of HWs, mainly in the group of health care professionals who directly care for patients with COVID-19 infection, due to their repeated exposure to the virus, can lead to result in higher SARS-CoV-2 viral load, and therefore worse clinical outcomes, including persistent OD, and whether these outcomes may be different from those in the general population; (ii) better quantify the alterations in smell associated with SARS-CoV-2 infection compared to other viral and respiratory infections; (iii) perform longitudinal studies to evaluate the presence and temporal duration of the OD, in people with both mild or moderate clinical conditions, as well as in severe processes, and to understand how persistent these disturbances are after the infection has been resolved; (iv) identify those patients with continued impairment who may need an olfactory-based training and analyze which training method could be more effective for this type of patient; (v) further study the close relationship between olfactory and taste dysfunction in patients with COVID-19, as some authors emphasize that they may represent an independent clinical entity.

To summarize, HWs are a population particularly at risk of infection because they have been on the frontline of COVID-19 management from the beginning of the epidemic. HWs with SARS-CoV-2 infection have a high prevalence of OD. Current evidence shows that OD is highly prevalent in COVID-19 infection, not only among HWs. Objective olfactory assessment methods are sensitive to the detection of long-lasting olfactory loss. Taking into account the high number of people infected in this pandemic and the significant proportion of cases of long-term olfactory loss perception, additional recapacitation of the health system will be necessary to offer treatment for smell and taste alterations in the subsequent recovery phase after COVID-19 infection.

The COVID-19 pandemic has exposed decades of underestimation of the olfactory senses, with limited availability for diagnostic tests and few therapeutic options.

## Figures and Tables

**Table 1:** Descriptive analysis per COVID-19 subgroups.

	No-COV	COV-without-Symp	COV-with-Symp		
Sample size	76	29	59		
	Mean (SD) or count	Mean (SD) or count	Mean (SD) or count	chi or F	*p*
Female	62	24	49	0.054	0.973
Age	48.3 (11.7)	43.3 (14.6)	44.7 (13)	2.235	0.11
Health hospital workers	44	21	44	4.706	0.095
Allergies	26	11	20	0.137	0.933
Other pathologies	24	10	14	2.273	0.321
None	58	20	54		
Allergies/hay fever	10	5	7		
High blood preasure	10	5	3		
Obesity	5	1	3		
Cardiac issues	0	1	0		
Neurological issues	1	1	0		
Respiratory issues	4	1	3		
Cancer	0	0	1		
Frequent smoking	25	4	6	11.154	0.003 **
Frequent alcohol consumption	7	7	4	5.99	0.06
Pre-COVID subjective smell performance	-	7.8 (1.58)	7.71 (1.46)	0.052	0.82
Post-COVID subjective smell performance	-				
Sniffin’ Sticks Olfactory Test scores					
Threshold	6.47 (2.6)	6.46 (2.56)	5.19 (3.56)	-	-
Discrimination	10.71 (2.71)	10.75 (2.29)	9.52 (3.12)	-	-
Identification–Free recall	2.82 (2.36)	2.76 (2.41)	1.88 (1.74)	-	-
Identification–Recognition	12.93 (2.14)	13.28 (2.55)	11.05 (3.68)	-	-
Identification–Subjective intensity	6.95 (1.42)	6.75 (1.46)	6.03 (2.39)	-	-
Total score	30.11 (4.83)	30.51 (4.39)	25.77 (8.46)	-	-

** *p* < 0.008.

**Table 2:** Olfactory unrelated symptomatology of COVID-19 patients.

Olfactory Unrelated COVID-19 Symtoms	COV-without (*n* = 29)	COV-with (*n* = 59)	chi	*p*
Asymptomatic	9	-	-	-
Headache	10	33	1.421	0.234
Muscle pain	9	29	1.923	0.166
Fatigue	11	28	0.552	0.457
Abdominal pain	1	13	3.727	0.053
Diarrhea	7	19	0.281	0.595
Respiratory issues	8	12	0.242	0.623
Chest oppression	4	10	0.005	0.944
Short of breath	8	12	0.242	0.623
Intense cough (dry or mucous)	7	25	2.061	0.151
Fever	3	7	0.001	0.999
Pharyngitis	5	6	0.36	0.548
Loss of appetite	4	22	4.082	0.043 *
Pneumonia	0	6	-	-
Olfactory related COVID-19 symptoms				
Olfactory loss or decrease	-	59		
Changes in food taste	-	43		
Stuffy nose	-	21		
Parosmia	-	26		
Phantosmia	-	7		

* *p* < 0.05.

**Table 3:** One-way Analysis of Covariance (ANCOVA) for each olfactory measure.

Olfactory Measure	Factor or Covariate	Mean Squared	df	F	*p*
Threshold	Age	72.2	1	8.762	0.003 **
	Smoking	7.798	1	0.946	0.332
	COVID	29.992	2	3.64	0.028 *
	COVID × Age	18.788	2	2.28	0.106
	COVID × Smoking	2.713	2	0.329	0.72
	Residuals	8.24	154		
Discrimination	Age	26.259	1	3.721	0.055
	Smoking	18.737	1	2.655	0.105
	COVID	25.48	2	3.611	0.029 *
	COVID × Age	2.845	2	0.403	0.669
	COVID × Smoking	8.74	2	1.239	0.292
	Residuals	7.057	154		
Identification–Free recall	Age	25.058	1	5.41	0.021 *
	Smoking	9.609	1	2.074	0.152
	COVID	15.303	2	3.307	0.039 *
	COVID × Age	1.068	2	0.231	0.794
	COVID × Smoking	2.957	2	0.638	0.529
	Residuals	4.632	154		
Identification-Recognition	Age	0.277	1	0.034	0.853
	Smoking	79.556	1	9.869	0.002 **
	COVID	54.908	2	6.811	0.001 **
	COVID × Age	0.136	2	0.017	0.983
	COVID × Smoking	1.834	2	0.227	0.797
	Residuals	8.062	154		
Identification–Subjective intensity	Age	23.914	1	7.606	0.006 **
	Smoking	10.192	1	3.242	0.073
	COVID	12.155	2	3.866	0.023 *
	COVID × Age	0.468	2	0.149	0.861
	COVID × Smoking	2.387	2	0.759	0.469
	Residuals	3.144	154		
Total	Age	200.155	1	5.281	0.011 *
	Smoking	257.297	1	6.789	0.022 *
	COVID	312.041	2	8.233	0.0004 **
	COVID × Age	38.205	2	1.008	0.367
	COVID × Smoking	30.605	2	0.807	0.447
	Residuals	37.899	154		

* *p* < 0.05 | ** *p* < 0.008.

**Table 4:** Percentage of success per item in Identification–Recognition score.

	COV-without-Symp	COV-with-Symp		
Item	%	%	chi	*p*
1 (Orange)	93.1	84.5	1.196	0.274
2 (Leather)	79.31	78.87	0.001	0.984
3 (Cinnamon)	68.96	76.05	0.071	0.791
4 (Mint)	100	95.77	0.878	0.349
5 (Banana)	89.65	83.1	0.617	0.432
6 (Lemon)	79.31	38.03	13.232	<0.0001 **
7 (Liquorice)	89.65	63.38	6.431	0.011 *
8 (Solvent)	75.86	53.52	3.923	0.047 *
9 (Garlic)	79.31	78.87	0.001	0.984
10 (Coffee)	79.31	57.74	3.818	0.051
11 (Apple)	65.51	49.29	1.915	0.166
12 (Clove)	72.41	66.19	0.295	0.587
13 (Pineapple)	75.86	63.38	1.284	0.257
14 (Rose)	100	84.5	4.427	0.034 *
15 (Anise)	86.2	69.2	2.898	0.088
16 (Fish)	93.11	92.95	0.002	0.963

* *p* < 0.05 | ** *p* < 0.008.

**Table 5:** Average scores per item in Identification–Subjective Intensity score.

	COV-without-Symp	COV-with-Symp			
Item	Mean (SD)	Mean (SD)	t	df	*p*
1 (Orange)	5.97	5.82	0.29	61.65	0.772
2 (Leather)	5.24	4.91	0.68	57.48	0.497
3 (Cinnamon)	6.04	5.24	1.26	52.43	0.213
4 (Mint)	8.48	7.09	3.44	89.54	0.0008 **
5 (Banana)	7.28	6.24	2.11	80.11	0.038 *
6 (Lemon)	5.45	4.64	1.65	59.59	0.104
7 (Liquorice)	6.03	5.08	1.87	68.84	0.066
8 (Solvent)	7.48	6.42	2.11	73.33	0.038 *
9 (Garlic)	8.17	7.26	2.06	88.64	0.042 *
10 (Coffee)	6.86	5.82	2.12	75.09	0.037 *
11 (Apple)	6.28	5.91	0.8	65.71	0.426
12 (Clove)	6.97	6.32	1.3	67.25	0.197
13 (Pineapple)	6.52	5.34	2.16	67.56	0.033 *
14 (Rose)	7.17	6.26	1.86	80	0.066
15 (Anise)	5.83	5.08	1.31	61.81	0.195
16 (Fish)	8.17	7.56	1.26	73.73	0.211

* *p* < 0.05 | ** *p* < 0.008.

## Data Availability

Data at individual level is available upon request to first author.

## Supplementary Materials

The following are available online: https://www.mdpi.com/article/10.3390/ijerph19095777/s1. Supplementary material S1: Visual Analog Scale (VAS) for recording odor’s subjective intensity in Identification (I) subtest; Supplementary material S2: Participants’ Flow Diagram.
