# Supplementary figures and images for: Long-Lasting Olfactory Dysfunction in Hospital Workers Due to COVID-19: Prevalence, Clinical Characteristics, and Most Affected Odorants
Source: Int J Environ Res Public Health. 2022 May 9;19(9):5777. doi: 10.3390/ijerph19095777 (PMC9105378; doi:10.3390/ijerph19095777)

[illegible]

1.- .....

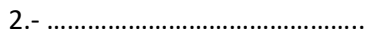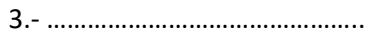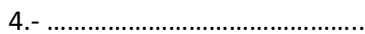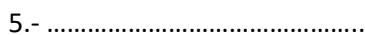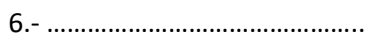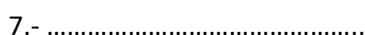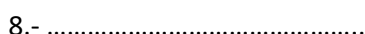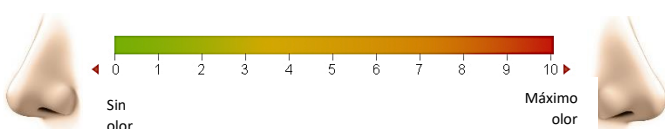

16.- .....

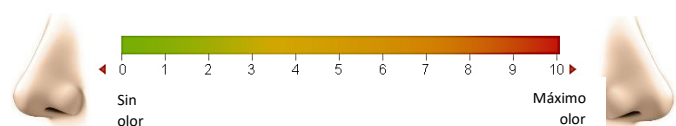

Supplement: Supplementary file 1 [file ijerph-19-05777-s001.zip › S1.pdf]

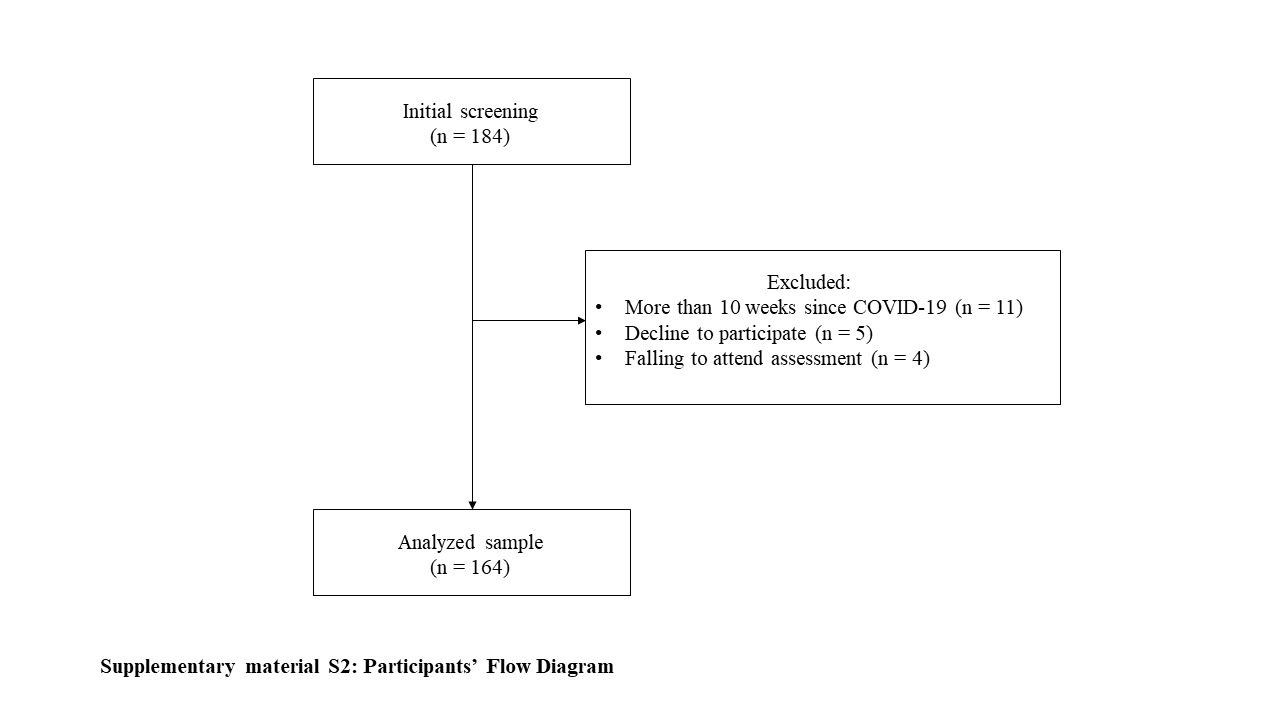

Supplement: Supplementary file 1 [file ijerph-19-05777-s001.zip › S2.jpg]
